# Digital Dilemma of Cyberbullying Victimization among High School Students: Prevalence, Risk Factors, and Associations with Stress and Mental Well-Being

**DOI:** 10.3390/children11060634

**Published:** 2024-05-24

**Authors:** Osama Mohamed Elsayed Ramadan, Majed Mowanes Alruwaili, Abeer Nuwayfi Alruwaili, Nadia Bassuoni Elsharkawy, Enas Mahrous Abdelaziz, Reda El Sayed El Badawy Ezzat, Eman Mahmoud Seif El-Nasr

**Affiliations:** 1 College of Nursing, Jouf University, Sakaka 72388, Saudi Arabia; omramadan@ju.edu.sa (O.M.E.R.); analrwili@ju.edu.sa (A.N.A.); nelsharkawy@ju.edu.sa (N.B.E.); emabdelhamid@ju.edu.sa (E.M.A.); 2 Maternal and Newborn Health Nursing Department, Faculty of Nursing, Cairo University, Cairo 11562, Egypt; 3 Psychiatric Mental Health Nursing Department, Faculty of Nursing, Cairo University, Cairo 11562, Egypt; 4 Department of Community Health Nursing, Faculty of Nursing, Zagazig University, Zagazig 44519, Egypt; rahassan@nursing.zu.edu.eg; 5 Department of Community Health Nursing, Faculty of Nursing, Cairo University, Cairo 11562, Egypt; emanseif@cu.edu.eg

**Keywords:** cyberbullying, high school students, mental well-being, perceived stress, risk factors

## Abstract

Cyberbullying has emerged as a pervasive problem among high school students, with potentially severe consequences for their mental well-being. This study aimed to investigate the prevalence, risk factors, and associations of cyberbullying with stress and mental well-being among high school students in Zagazig, Egypt. A cross-sectional study was conducted among 562 high school students using a random sampling technique. The data were collected using a self-administered questionnaire that included the Cyberbullying Scale, Perceived Stress Scale (PSS-10), and General Health Questionnaire (GHQ-12). Descriptive statistics, independent samples *t*-tests, multiple regression, mediation, and logistic regression analyses were employed for data analysis. The prevalence of cyberbullying victimization was 38.3%, with 20.6% exposed to two or three cyberbullying behaviors and 4.1% exposed to four or more. Female students, those under 18 years old, those with lower educational achievement, and those with higher daily internet use were more likely to experience cyberbullying. Cyberbullied students reported significantly higher levels of perceived stress and poorer mental well-being compared to non-cyberbullied students. Perceived stress likely mediated the relationship between cyberbullying victimization and general psychological health. Cyberbullying is a significant problem among high school students in Zagazig, Egypt, with detrimental effects on their stress levels and mental well-being. Targeted interventions and prevention strategies are needed to address cyberbullying and promote the well-being of adolescents in the digital age.

## 1. Introduction

In the rapidly evolving digital landscape, cyberbullying has emerged as a pervasive and alarming phenomenon, particularly among high school students [1,2,3,4,5]. As adolescents spend more time connected through social media and online platforms, they become more vulnerable to this new form of aggression [6,7,8,9]. Cyberbullying, defined as the use of electronic communication technologies to engage in repeated and intentional hostile behavior toward others [10,11,12], transcends the boundaries of traditional bullying. It can occur relentlessly, infiltrating the lives of victims even in the perceived safety of their own homes, leaving them feeling trapped and powerless [13,14]. The ubiquitous nature of cyberbullying and its potential for severe psychological consequences have raised serious concerns among educators, mental health professionals, and parents, emphasizing the critical need for a comprehensive understanding of this digital dilemma [15,16,17].

Numerous studies have investigated cyberbullying among high school students, examining its prevalence, risk factors, and consequences [18,19,20,21,22]. However, there is a need for more research in diverse cultural contexts, such as Egypt, and a deeper examination of the specific risk factors and associations with stress and mental well-being.

The need for more research on cyberbullying in diverse cultural contexts, such as Egypt, stems from the potential differences in the prevalence, nature, and consequences of cyberbullying across various societies. Cultural norms, values, and social structures may influence the way cyberbullying is perceived, experienced, and addressed [23,24,25]. For instance, in Egypt, where traditional gender roles and collectivistic values are prevalent, the impact of cyberbullying on adolescents’ mental health and well-being may differ from that in Western, individualistic cultures [26]. Additionally, the rapidly increasing access to technology and the internet in Egypt, coupled with the limited awareness and prevention programs, may contribute to a higher risk of cyberbullying among the Egyptian youth [27,28]. Understanding these cultural nuances is crucial for developing effective, context-specific interventions and policies to combat cyberbullying and its negative consequences [29,30,31].

The prevalence of cyberbullying victimization among adolescents in Egypt has been explored in several studies, revealing rates ranging from 27.4% to 83.25% [23,32,33,34]. These findings highlight the importance of investigating cyberbullying experiences and their associated factors within the Egyptian context. 

To develop effective prevention and intervention strategies, it is crucial to identify the risk factors associated with cyberbullying among high school students [35,36,37]. While some studies suggest that girls are more likely to experience cyberbullying [38,39], others have found no significant gender differences [40,41,42]. The role of age remains inconclusive, with inconsistent findings regarding the peak of victimization during adolescence [43,44,45,46]. Socioeconomic status and race/ethnicity have also yielded mixed results, highlighting the need for further investigation [47,48]. Online risk factors, such as increased time spent on social networks and risky online behaviors, have consistently been associated with higher rates of victimization by means of cyberbullying [49,50]. Furthermore, psychosocial factors, including low self-esteem, poor social skills, and previous victimization experiences, have emerged as significant predictors [51,52].

The impact of cyberbullying on the mental health and well-being of high school students cannot be overstated [53,54,55,56,57]. Adolescence is a critical developmental period marked by increased vulnerability to social and emotional stressors [58,59]. Cyberbullying during this sensitive stage can have profound and lasting effects on psychological functioning, academic performance, and overall quality of life [60,61]. Numerous studies have consistently linked cyberbullying to higher levels of stress, depression, anxiety, and suicidal ideation among adolescents [60,62,63]. These mental health challenges can lead to a cascade of negative outcomes, such as avoidance of school, substance abuse, and interpersonal difficulties [18,59,61].

Addressing cyberbullying in high school is of paramount importance, particularly in the Egyptian context, where it can have severe consequences for adolescents’ mental health, academic performance, and overall well-being. The insights gained from the research can inform the development and implementation of evidence-based educational policies, prevention programs, and mental health interventions tailored to the specific needs and challenges of the Egyptian youth. By understanding the prevalence, risk factors, and mental health correlates of cyberbullying, schools can develop targeted initiatives to raise awareness, promote digital citizenship, and foster a culture of kindness and respect both online and offline. Furthermore, identifying effective strategies to support students who have experienced cyberbullying is crucial to mitigate its negative impact on their well-being and create a more inclusive school environment [64,65,66,67].

Despite the growing body of research on cyberbullying, significant gaps remain in our understanding of this phenomenon, particularly in the context of high school environments [68,69]. The variability in prevalence rates across studies can be largely attributed to differences in measures, study populations, instructions, and the timing of the research [70,71,72]. To address this variability and provide a more accurate understanding of the prevalence of cyberbullying, it is essential to use standardized measures and clearly define the study context, population, and time frame [73]. Furthermore, while several risk factors have been identified, such as gender, time spent online, and prior victimization [39,40,53], the relative contributions and complex interactions of these factors in the context of high school cyberbullying remain understudied [74]. The current study aims to contribute to understanding the prevalence of cyberbullying and its associated factors in the specific context of high school students in Zagazig, Egypt, using well-defined measures and a clear timeframe, thus addressing the need for more context-specific research in this field.

Based on the reviewed literature and the identified gaps in our understanding of cyberbullying in the Egyptian context, the current study aims to address the following research questions: (1) What is the prevalence of cyberbullying among high school students in Egypt? This question is informed by the limited and varying prevalence rates reported in previous studies conducted in Egypt, highlighting the need for a more comprehensive assessment using standardized measures and a clear timeframe. (2) Is there a significant relationship between cyberbullying victimization and high school students’ mental well-being and perceived stress levels? This question stems from the consistent evidence linking cyberbullying to adverse mental health outcomes among adolescents but remains underexplored in the Egyptian context. (3) Is perceived stress mediating the relationship between cyberbullying victimization and general psychological health? This question is motivated by the need to understand the mechanisms underlying the impact of cyberbullying on mental well-being, especially the role of stress as a potential mediator, which has not been extensively examined in previous research.

In conclusion, the current study aims to fill the gaps in our understanding of cyberbullying among Egyptian high school students by addressing the aforementioned research questions. By providing insights into the prevalence, risk factors, and associations with stress and mental well-being, this study contributes to the development of evidence-based interventions and policies tailored to the Egyptian context. Ultimately, this study represents a crucial step toward promoting the overall health and well-being of Egyptian adolescents in the digital age. Cyberbullying among high school students is a pressing problem that demands urgent attention from researchers, educators, and policymakers [68,71,72,75]. The current study aims to contribute to our understanding of the prevalence of cyberbullying and its associated factors in the specific context of high school students in Zagazig, Egypt, a region representative of urban adolescent populations in the country, using well-defined measures and a clear timeframe, thus addressing the need for more context-specific research in this field. Investing in research and action to address cyberbullying in high school is not only a matter of protecting individual students but also a critical step in promoting the overall health and success of future generations in an increasingly digital world.

## 2. Materials and Methods

### 2.1. Design

A cross-sectional research design was employed in this study. This design allows for data collection from a large sample at a single point in time, providing a snapshot of the current state of the phenomenon under investigation [76]. The cross-sectional design is well suited for examining the complex interplay between variables and generating hypotheses for future research while also offering practical insights for the development of targeted prevention and intervention strategies [77]. This design has been widely used in previous studies investigating cyberbullying and its correlates among adolescents [49,51,65,71,78], demonstrating its suitability for the current research objectives.

### 2.2. Sample

The study sample comprised 562 high school students (266 males and 296 females) from the western sector of Zagazig governorate, Egypt. The participants were enrolled in the 10th, 11th, and 12th grades, corresponding to the 1st, 2nd, and 3rd grades of the Egyptian high school system. Their ages ranged from 14 to 19 years, with a mean of 16.5 (SD = 1.2). Educational achievement and financial level were measured on a 5-point scale, where 1 indicated poor status, and 5 indicated excellent status. The mean educational achievement score was 3.8 (SD = 1.1), while the average financial level was 3.9 (SD = 0.9). Regarding internet usage, the participants reported spending an average of 3.2 h (SD = 1.8) online daily.

To qualify for participation, students were required to own a smartphone and have regular access to the internet. We excluded students with chronic diseases, physical disabilities, or neuropsychiatric disorders to maintain a homogeneous health status across the sample. The selected grade levels represent a critical developmental period where adolescents are increasingly engaged with digital technologies and social networks, heightening their vulnerability to cyberbullying [35,63,75,79]. This focus also allows for an in-depth examination of the impact of cyberbullying on mental health during a phase characterized by increased social pressures and peer influence.

The sample size was initially calculated using the Raosoft sample size calculator (Raosoft Inc., Seattle, WA, USA), with a confidence level of 95% and a margin of error of 5%, to ensure robustness and reliability in the findings [80]. Based on these parameters, the target sample size was determined to be 382 students. To obtain a representative sample encompassing all demographic groups, we adopted a random sampling design. Three schools were randomly selected from the list of thirteen high schools in the western sector of the Zagazig governorate. Within each selected school, classes were randomly chosen based on predetermined criteria to ensure a balanced representation of different grade levels. All students in the selected classes were invited to participate, allowing for the inclusion of a diverse range of high school students with varying grade levels, educational institutions, and demographic characteristics. The random selection process at both school and class levels minimized potential selection bias, enhancing the sample’s representativeness. The study survey was distributed to 600 students, ultimately receiving responses from 562 participants, resulting in a completion rate of 93.7% (562/600). This high level of participation and completion can be attributed to the clear instructions provided by the research assistants and the controlled classroom environment, which encouraged students to engage fully in the survey.

### 2.3. Data Collection Tools

The data were collected using a self-administered questionnaire, which consisted of the following sections:

1. Socio-demographic Characteristics:

This section gathered information on participants’ socio-demographic characteristics, such as age, sex, educational achievements, financial levels, and daily internet hours. These variables are essential to understanding the context and potential influencing factors related to cyberbullying experiences.

2. Cyberbullying Scale:

The Cyberbullying Scale developed by Qudah et al. (2020) [81] was selected for this study due to its cultural relevance, comprehensive assessment of various cyberbullying dimensions, and demonstrated psychometric properties in an Arabic-speaking population. These factors make it an appropriate tool for investigating cyberbullying experiences among Egyptian high school students. The scale consists of 26 items classified into 5 dimensions: mockery and defamation (8 items), ridicule and threats (3 items), invasion of privacy (5 items), exclusion from group activities (5 items), and sexual harassment (5 items). Participants responded to each item using a 5-point Likert scale, ranging from 1 (strongly disagree) to 5 (strongly agree), indicating their experiences with cyberbullying in the past six months [82,83]. The original scale demonstrated good internal consistency, with Cronbach’s alpha values ranging from 0.74 to 0.93 [84]. In the current study, the scale’s reliability was assessed using Cronbach’s alpha, yielding a value of 0.84, indicating satisfactory internal consistency.

3. General Psychological Health Questionnaire (GHQ-12):

The GHQ-12, developed by David Goldberg (1970) [85] and previously used with Arabic samples by El-Rufaie and Daradkeh [86], was used to assess the severity of the psychological symptoms of the participants. The Arabic version of the GHQ-12 proved to be reliable, as shown by Cronbach’s alpha of 86. The items were rated on a 4-point Likert scale (0–3), with a cut-off score of 4 or higher indicating psychological impairment. Participants who scored less than 4 were classified as having “normal” mental health, while those who scored 4 or more were considered a “risk group for mental problems”. The GHQ-12 has been widely used in healthcare research and has demonstrated good validity in assessing psychological distress [87,88,89]. In the current study, the questionnaire exhibited satisfactory internal consistency, with a Cronbach’s alpha value of 0.844.

4. Perceived Stress Scale (PSS-10):

The PSS-10, developed by Cohen and Williamson (1988) [90,91] and translated into Arabic by Monique Chaaya et al. (2010) [92], was used to assess participants’ perceived stress levels. The scale consists of 10 items that measure the degree to which respondents find their lives unpredictable, uncontrollable, and overloaded. Each item is rated on a 5-point Likert scale (0 = never, 4 = very often). The PSS-10 includes six positively worded items (items 1, 2, 3, 6, 9, and 10: positive factor) and 4 negatively worded items (items 4, 5, 7, and 8: negative factor). Negatively worded items were reverse coded during analysis, with higher scores indicating higher stress levels. The PSS-10 has been widely used and validated in various populations and languages [90,93]. Cronbach’s alpha for assessing the internal consistency and reliability of the original Arabic PSS-10 was 0.74 [92]. In the current study, the scale demonstrated satisfactory internal consistency, with a Cronbach’s alpha value of 0.80.

### 2.4. Ethical Approval

Ethical considerations were of the utmost importance throughout the research process. The current study obtained ethical approval from the Research Ethics Committee of the Faculty of Nursing of Zagazig University, Zagazig, Egypt, under reference number 63, in September 2023, ensuring adherence to ethical principles and guidelines. Official permission was also obtained from the educational director of the western sector of Zagazig governorate. Before administering the questionnaire, the researchers emphasized the voluntary nature of participation and provided comprehensive information on the purpose and objectives of the study. Informed consent was obtained from the participants, and in the case of minors, from both participants and their parents or guardians. Researchers prioritized the protection of the confidentiality and anonymity of participants, treating all data with strict confidentiality and using them solely for research purposes. By obtaining the necessary approvals, following informed consent procedures, and implementing measures to protect the participants’ privacy, this study demonstrates a strong commitment to ethical research practices, improving its credibility and trustworthiness.

### 2.5. Procedure

This study was conducted in Zagazig, a city in the Sharqia Governorate of Lower Egypt, approximately 80 km northeast of Cairo. Data collection took place from 1 October 2023 to 15 January 2024 in the western sector of the Zagazig governorate, which includes 13 high schools with a diverse student population of approximately 54,279. The researchers explained the study’s aims to all eligible students and distributed the questionnaire based on the class registers. This approach ensured that all eligible students had the same opportunity to participate and minimized the risk of selection bias. The participating adolescents were asked to complete an anonymous self-report questionnaire in the classroom during school hours to ensure confidentiality and minimize potential reporting bias. The classroom setting provided a controlled environment, and the presence of trained research assistants allowed for proper supervision and support throughout the data collection process.

Completing the questionnaire took approximately 15–20 min, ensuring that the study did not significantly disrupt the students’ regular school activities. The research assistants were readily available to clarify any questions or concerns that the participants might have had while filling out the questionnaire. As detailed in Section 2.2, 562 out of the 600 students approached participated in this study and provided complete responses to the questionnaire, resulting in a high completion rate of 93.7%. After completing the questionnaires, the data were carefully codified to facilitate statistical analysis. This process involved assigning numerical codes to the responses, ensuring that the data were properly organized and ready for further analysis using appropriate statistical software.

### 2.6. Statistical Analysis

The collected data were analyzed using the Statistical Package for Social Sciences (SPSS) version 26.0. Descriptive statistics, including frequencies, percentages, means, and standard deviations, were employed to characterize the socio-demographic attributes, cyberbullying experiences, perceived stress levels, and general psychological health of the study participants. Independent samples *t*-tests were utilized to compare the means of continuous variables such as age, educational achievement, financial level, daily internet use, perceived stress, and general psychological health between cyberbullied and non-cyberbullied students. The prevalence and extent of cyberbullying victimization were determined using descriptive statistics. A multiple linear regression analysis using the forced entry method was conducted to identify significant predictors of cyberbullying victimization among high school students. All predictor variables (sex, age, educational achievement, and daily internet use) were entered simultaneously into the model, allowing for an assessment of each variable’s unique contribution to the outcome while controlling for the other predictors. Separate linear regression analyses were conducted to examine the relationships between cyberbullying victimization, perceived stress, and general psychological health. In each model, cyberbullying victimization served as the predictor variable, while perceived stress and general psychological health were the outcome variables.

A simple mediation analysis (Model 4) was conducted using the PROCESS macro for SPSS to investigate the role of perceived stress in the relationship between cyberbullying victimization and general psychological health. In this model, cyberbullying victimization served as the predictor variable (X), perceived stress as the mediator (M), and general psychological health as the outcome variable (Y). All statistical tests were two-tailed, and a *p*-value less than 0.05 was considered statistically significant. The results of these analyses are presented in the corresponding sections of the results.

## 3. Results

Table 1 provides an overview of the key socio-demographic characteristics of the study sample, including information on the distribution of sex, age, educational achievement, financial level, and daily use of the Internet. The descriptive statistics presented in this table help contextualize the sample and facilitate the interpretation of the study findings.

Table 1 presents the socio-demographic characteristics of the study sample (*n* = 562). The sample consisted of 266 males (47.3%) and 296 females (52.7%). The mean age of the participants was 16.5 years (SD = 1.2). Regarding educational achievement, measured on a 5-point scale (1 = poor, 5 = excellent), the mean score was 3.8 (SD = 1.1). For the financial level, also measured on a 5-point scale (1 = poor, 5 = excellent), the mean score was 3.9 (SD = 0.9). On average, participants reported using the Internet for 3.2 h per day (SD = 1.8).

Table 2 details the prevalence and extent of cyberbullying among the study participants, categorizing the victimized students by the frequency of their experiences. This classification highlights the severity and distribution of cyberbullying within the sample.

Table 2 presents the cyberbullying victimization status and frequency for the study sample (*n* = 562). The majority of the participants (61.7%) reported not being cyberbullied, while 38.3% experienced cyberbullying victimization. Among those who were cyberbullied (*n* = 215), 13.5% were exposed to one cyberbullying behavior, 20.6% experienced two or three behaviors, and 4.1% were exposed to four or more cyberbullying behaviors.

Table 3 compares the socio-demographic characteristics of participants who reported being victims of cyberbullying (*n* = 215) and those who did not (*n* = 347). Independent samples *t*-tests were conducted to examine the differences between the two groups across age, educational achievement, financial level, and daily internet use.

The results indicated in Table 3 show significant differences between cyberbullied and non-cyberbullied participants across all socio-demographic variables examined. Cyberbullied participants were younger, had lower educational achievement and financial levels, and spent more time on the Internet daily compared to their non-cyberbullied counterparts. These findings suggest that certain socio-demographic factors may be associated with an increased risk of experiencing cyberbullying victimization.

A multiple linear regression analysis was conducted to examine the predictors of cyberbullying victimization, with the results presented in Table 4. The model included sex, age, educational achievement, and daily internet use as predictor variables.

The regression analysis in Table 4 reveals that being female, being younger, having lower educational achievement levels, and spending more time on the Internet daily were significant predictors of cyberbullying victimization. Educational achievement had the strongest influence on the likelihood of experiencing cyberbullying. These findings highlight the importance of considering socio-demographic factors when developing targeted interventions to prevent and address cyberbullying.

The relationships between cyberbullying victimization, perceived stress, and general psychological health were examined using separate linear regression analyses, with the results presented in Table 5. Cyberbullying victimization served as the predictor variable, while perceived stress and general psychological health were the outcome variables.

The regression analyses in Table 5 reveal significant associations between cyberbullying victimization, perceived stress, and general psychological health. Higher levels of cyberbullying victimization are associated with increased perceived stress and poorer general psychological health, with large effect sizes observed for both outcomes. These findings underscore the substantial impact of cyberbullying on students’ mental well-being and highlight the need for interventions that address the psychological consequences of victimization.

A mediation analysis was conducted to investigate the role of perceived stress in the relationship between cyberbullying victimization and general psychological health, with the results presented in Table 6. The analysis was performed using the PROCESS macro for SPSS, with cyberbullying victimization as the predictor variable, perceived stress as the mediator, and general psychological health as the outcome variable.

**Figure 1:**
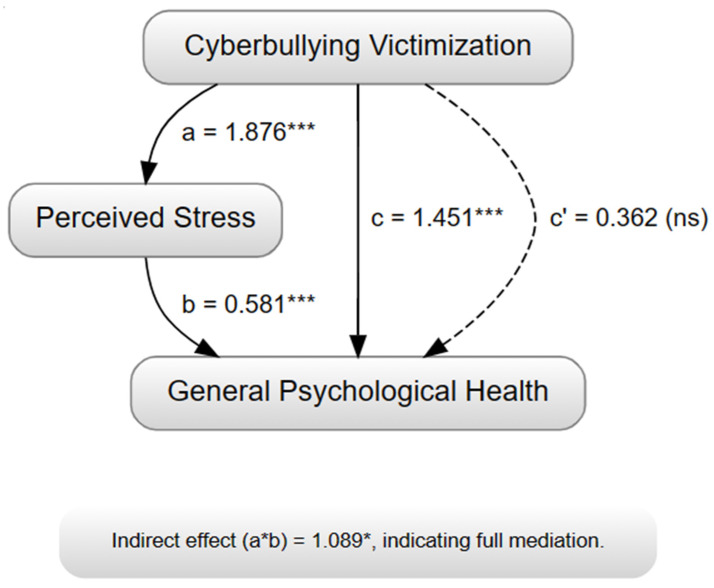
Mediation model illustrating impact of cyberbullying victimization on general psychological health through perceived stress.

The mediation analysis (Table 6 and Figure 1) reveals that perceived stress significantly mediates the relationship between cyberbullying victimization and general psychological health. The significant indirect effect suggests that victimization through cyberbullying influences general psychological health through its impact on perceived stress. These findings highlight the importance of addressing stress management and coping strategies in interventions aimed at mitigating the negative consequences of cyberbullying on mental well-being. Figure 1 presents the simple mediation model tested in the current study, depicting the relationships between cyberbullying victimization, perceived stress, and general psychological health. The paths labeled a, b, c, and c’ correspond to the coefficients reported in Table 6.

Figure 1. Mediation Model Illustrating the Impact of Cyberbullying Victimization on General Psychological Health through Perceived Stress. Solid arrows represent significant paths (*p* < 0.001). The dashed arrow represents a non-significant direct path after controlling for the mediator. Path a represents the effect of cyberbullying victimization on perceived stress. Path b represents the effect of perceived stress on general psychological health. Path c represents the total effect of cyberbullying victimization on general psychological health, without controlling for the mediator (perceived stress). Path c’ represents the direct effect of cyberbullying victimization on general psychological health, after controlling for the mediator. The indirect effect (a*b) represents the mediated effect of cyberbullying victimization on general psychological health through perceived stress. *** *p* < 0.001, * *p* < 0.05, ns = not significant.

## 4. Discussion

The current study aimed to investigate the prevalence, risk factors, and associations of victimization by means of cyberbullying with stress and mental well-being among high school students in Zagazig, Egypt. The findings provide valuable insights into the extent and nature of cyberbullying experienced by this population, as well as the significant psychological consequences associated with victimization.

The prevalence of cyberbullying victimization among high school students in this study was found to be substantial, with 38.3% of the sample reported having experienced cyberbullying. This finding is consistent with previous research that has reported high prevalence rates of cyberbullying among adolescents [5,15]. The prevalence rate in the current study falls within the range of estimates reported in the literature, highlighting the widespread nature of this problem among high school students in Egypt. Furthermore, this study revealed that 20.6% of the students were exposed to two or three cyberbullying behaviors, and 4.1% were exposed to four or more, underscoring the varying degrees of victimization experienced by the students. These results emphasize the need for comprehensive interventions to address the various manifestations of cyberbullying [94,95,96,97].

The high prevalence of cyberbullying victimization found in our study aligns with previous research carried out in the Egyptian context [23,32,33]. The current study found that female students reported higher rates of cyberbullying victimization compared to their male counterparts. However, it is important to consider that the Cyberbullying Scale used in this study included items related to sexual harassment, which may have contributed to the higher victimization rates among women. Previous research in the Egyptian context has highlighted the prevalence of sexual harassment as a form of cyberbullying experienced by female adolescents and young adults [23,33]. While our findings suggest that gender differences in cyberbullying victimization exist, the inclusion of sexual harassment in the assessment may limit the generalizability of this result to other forms of cyberbullying. Future studies should investigate gender differences in specific types of cyberbullying experiences to provide a more comprehensive understanding of the relationship between gender and cyberbullying victimization.

This finding underscores the need to consider the specific social and cultural factors that can influence cyberbullying experiences among Egyptian adolescents. For example, the rapid expansion of internet access and social media use in Egypt, coupled with the lack of comprehensive cyberbullying prevention programs and legal consequences, may contribute to the high rates of victimization [34]. Additionally, gender roles and expectations in Egyptian society may play a role in the higher prevalence of cyberbullying among female students, particularly in the form of sexual harassment [23,32,33,34]. Future research should further explore how these social and cultural factors interact with individual and technological risk factors to shape cyberbullying experiences in the Egyptian context. Understanding these unique dynamics can inform the development of culturally sensitive prevention and intervention strategies tailored to the needs of Egyptian high school students.

The multiple regression analysis identified several significant predictors of cyberbullying victimization among high school students, including the female gender, younger age, lower educational achievement, and higher daily use of the Internet. These findings are consistent with previous research that has identified demographic, academic, and internet usage factors as important predictors of cyberbullying experiences [98,99,100,101]. The identification of these risk factors has important implications for the development of targeted prevention and intervention strategies [38,102]. Schools and educators can use this information to identify students who may be more vulnerable to cyberbullying and provide them with appropriate support and resources [103,104]. The strong association between poor educational performance and cyberbullying highlights the importance of addressing academic difficulties and providing educational support to students as a means of reducing their vulnerability to online victimization [105,106].

Although the current study did not directly investigate the relationship between cyberbullying victimization and specific family environmental factors such as single-parent households or low-income families, our findings did reveal a significant association between lower financial level and increased risk of cyberbullying victimization (Table 3). This suggests that socioeconomic status may play a role in shaping adolescents’ vulnerability to cyberbullying. Previous research has indicated that youths from low-income families may be at higher risk of experiencing cyberbullying due to factors such as limited parental supervision, reduced access to resources and support, and increased exposure to online risks [5,107,108,109]. Additionally, single-parent families may face unique challenges in providing adequate monitoring and support for their children’s online activities, potentially increasing the risk of cyberbullying involvement [110]. While the current study provides initial evidence of the association between financial level and cyberbullying victimization, future research should further explore the specific mechanisms through which family environmental factors, such as single-parent households and low-income status, may influence cyberbullying experiences among Egyptian adolescents. Understanding these relationships can inform the development of targeted interventions and support systems for youths from diverse family backgrounds.

The comparison of perceived stress and general psychological health between cyberbullied and non-cyberbullied students revealed significant differences between the two groups. Cyberbullied students reported significantly higher levels of perceived stress and poorer general psychological health compared to non-cyberbullied students [111,112]. These findings are consistent with previous research that has documented the negative psychological consequences of victimization by means of cyberbullying, including increased stress, depression, anxiety, and suicidal ideation [113,114]. Strong associations between cyberbullying victimization, increased perceived stress, and poorer general psychological health underscore the need for effective interventions and support services to address the mental health consequences of cyberbullying [115,116].

The linear regression analyses revealed significant associations between cyberbullying victimization, perceived stress, and general psychological health. These findings suggest that higher levels of cyberbullying victimization are predictive of increased perceived stress and poorer general psychological health among high school students. The substantial effect sizes observed for both outcomes underscore the magnitude of the impact that cyberbullying victimization can have on adolescents’ mental well-being. These results can be interpreted through the lens of the transactional theory of stress and coping [117], which posits that individuals’ appraisals of stressful events, such as cyberbullying experiences, influence their emotional and behavioral responses. When cyberbullying is perceived as a significant threat or challenge, it may overwhelm students’ coping resources, leading to heightened stress levels and psychological distress [118]. Moreover, the chronic and pervasive nature of cyberbullying may contribute to a sense of helplessness and diminished self-esteem, further exacerbating the negative impact on mental health [119,120]. The linear regression findings emphasize the need for interventions that not only target cyberbullying behaviors but also equip students with effective stress management and coping strategies to mitigate the adverse psychological consequences of victimization.

The mediation analysis revealed that perceived stress significantly mediates the relationship between cyberbullying victimization and general psychological health. As illustrated in Figure 1, the mediation analysis provided evidence for the role of perceived stress in explaining the relationship between cyberbullying victimization and general psychological health. The significant indirect effect suggests that cyberbullying victimization leads to increased perceived stress, which, in turn, contributes to poorer general psychological health. This finding highlights the interconnected nature of these variables and the cascading effect that cyberbullying can have on the mental well-being of high school students [121,122,123,124,125]. The mediation analysis provides valuable insights into the mechanisms underlying the relationship between cyberbullying victimization and general psychological health. The results emphasize the importance of addressing cyberbullying not only as an isolated issue but also as a factor that can contribute to increased stress levels and psychological problems among adolescents [126,127]. This finding underscores the need for interventions that target both cyberbullying and its associated psychological consequences, with a specific focus on stress management and coping strategies. By addressing perceived stress, interventions may effectively buffer the detrimental effects of cyberbullying victimization on adolescents’ mental well-being.

Logistic regression analysis further identified lower educational achievement, cyberbullying victimization, and increased perceived stress as significant predictors of the risk of poor general psychological health. These results corroborate the findings of the mediation analysis and highlight the complex interplay between cyberbullying, stress, and mental health outcomes. Future research should explore the long-term consequences of cyberbullying victimization and perceived stress on adolescents’ psychological well-being, taking into account potential moderating factors such as social support, resilience, and coping mechanisms. A better understanding of these relationships can inform the development of comprehensive intervention programs that effectively mitigate the negative impact of cyberbullying on students’ mental health.

While our study identifies several risk factors associated with cyberbullying victimization, such as gender, age, educational achievement, and internet use, it is important to acknowledge that these factors may not represent direct causes of cyberbullying. Rather, they may contribute to an individual’s vulnerability to experiencing cyberbullying. Future research employing qualitative methods or longitudinal designs could provide deeper insights into the specific causes and motivations behind cyberbullying perpetration.

### Practical Implications and Future Directions

The findings of this study have important practical implications for educators, mental health professionals, and policymakers. The high prevalence of cyberbullying among high school students in Egypt underscores the need for comprehensive prevention and intervention programs that address the various forms and dimensions of cyberbullying. These programs should aim to raise awareness about the different types of cyberbullying, promote responsible use of the Internet and digital literacy skills, and provide support and resources for students who have experienced victimization. Given the gender differences in cyberbullying experiences, it is crucial to develop gender-specific interventions and support services, particularly to address the victimization of sexual harassment among female students. Schools and educators must prioritize creating a safe and supportive environment that encourages the reporting of cyberbullying incidents and provides appropriate support and resources for victims.

The significant associations between cyberbullying, perceived stress, and psychological well-being emphasize the importance of integrating mental health support and stress management strategies into cyberbullying prevention and intervention programs. Mental health professionals must work closely with schools to provide accessible and effective support services to students who have experienced cyberbullying. This may include individual counseling, group therapy, and psychoeducation on coping strategies and building resilience.

Future research should build on the findings of this study by examining the long-term effects of cyberbullying on the mental health and well-being of high school students. Longitudinal studies can provide valuable information on the trajectories of psychological distress and the factors that contribute to resilience among cyberbullying victims. Furthermore, qualitative research methods, such as in-depth interviews and focus groups, can offer a more nuanced understanding of the lived experiences of cyberbullying victims and the coping strategies they employ.

Future studies should also investigate the effectiveness of various prevention and intervention strategies, including school programs, online safety education, and mental health support services, in reducing the incidence and impact of cyberbullying among high school students. Evaluating the efficacy of these interventions can inform evidence-based practices and guide the development of comprehensive and effective approaches to combating cyberbullying. Additionally, research should explore the role of bystanders in cyberbullying incidents and identify effective strategies to encourage bystander intervention and reporting. Empowering students to take an active role in preventing and addressing cyberbullying can contribute to creating a more positive and supportive school environment.

Addressing the complex issue of cyberbullying requires a multi-faceted approach that involves the cooperation of educators, mental health professionals, parents, and policymakers. By raising awareness, developing effective prevention and intervention strategies, and fostering a supportive and inclusive school environment, we can work toward creating safer online and offline spaces for adolescents to thrive and reach their full potential.

## 5. Conclusions

In conclusion, this study provides a comprehensive examination of the prevalence, risk factors, and associations of cyberbullying victimization with stress and mental well-being among high school students in Zagazig, Egypt. The findings highlight the widespread nature of cyberbullying in this population and the significant psychological consequences associated with victimization. The identification of specific risk factors, such as gender, age, educational achievement, and internet use, provides valuable information for the development of targeted prevention and intervention strategies. The present study also underscores the need for gender-specific approaches to address cyberbullying, especially in the context of sexual harassment experienced by female students.

Strong associations between victimization by means of cyberbullying, perceived stress, and psychological well-being emphasize the importance of a holistic approach to addressing cyberbullying, one that integrates mental health support and stress management strategies. Mental health professionals and educators must work collaboratively to provide comprehensive support services for students who have experienced cyberbullying.

This study contributes to the growing body of research on cyberbullying and its impact on the mental well-being of adolescents. The findings provide a foundation for future research and practical interventions aimed at combating cyberbullying and promoting the well-being of high school students in the digital age. By addressing the prevalence, risk factors, and psychological consequences of cyberbullying, we can work toward creating a safer and more supportive environment for adolescents, both online and offline.

## Figures and Tables

**Table 1:** Socio-demographic characteristics of the study sample (*n* = 562).

Variable	*n*	%	Mean ± SD
Sex:			
Male	266	47.3%	
Female	296	52.7%	
Age (years):			16.5 ± 1.2
Educational Achievement (1–5 scale):			3.8 ± 1.1
Financial Level (1–5 scale):			3.9 ± 0.9
Daily Internet Use (hours):			3.2 ± 1.8

Note: educational achievement and financial level were measured on a 5-point scale (1 = poor, 5 = excellent).

**Table 2:** Prevalence and extent of cyberbullying victimization (*n* = 562).

Variable	*n*	%
Cyberbullying Victimization Status:		
Not bullied	347	61.7%
Bullied	215	38.3%
Frequency of Cyberbullying Victimization:		
Exposed to one behavior	76	13.5%
Exposed to two or three behaviors	116	20.6%
Exposed to four or more behaviors	23	4.1%

Note: The frequency of cyberbullying victimization data is presented only for the bullied group (*n* = 215). Participants in the “Not Bullied” group (*n* = 347) did not report any cyberbullying experiences.

**Table 3:** Comparison of socio-demographic characteristics by cyberbullying victimization status.

Variable	Not Bullied (*n* = 347)	Bullied (*n* = 215)	t	*p*-Value
Age (years)	16.6 ± 1.2	16.3 ± 1.1	2.98	0.003 **
Educational Achievement (1–5)	4.1 ± 0.9	3.3 ± 1.1	9.58	<0.001 ***
Financial Level (1–5)	4.0 ± 0.9	3.8 ± 1.0	2.44	0.015 *
Daily Internet Use (hours)	2.9 ± 1.6	3.7 ± 1.9	−5.35	<0.001 ***

Note: * *p* < 0.05, ** *p* < 0.01, and *** *p* < 0.001.

**Table 4:** Multiple regression analysis predicting cyberbullying victimization (*n* = 562).

Predictor Variables	B (95% CI)	SE	β	t	*p*-Value
Sex (Female)	0.152 (0.012, 0.292)	0.071	0.081	2.135	0.033 *
Age	−0.079 (−0.138, −0.021)	0.030	−0.102	−2.673	0.008 **
Educational Achievement	−0.201 (−0.262, −0.140)	0.031	−0.254	−6.474	<0.001 ***
Daily Internet Use (hours)	0.106 (0.066, 0.146)	0.020	0.193	5.199	<0.001 ***

Note: A multiple linear regression analysis using the forced entry method was conducted. B = unstandardized regression coefficient; SE = standard error; β = standardized regression coefficient; CI = confidence interval. R^2^ = 0.178, F (4, 557) = 30.16, and *p* < 0.001. * *p* < 0.05, ** *p* < 0.01, and *** *p* < 0.001.

**Table 5:** Regression analyses of perceived stress and general psychological health on cyberbullying victimization (*n* = 562).

Variables	B (95% CI)	SE	t	*p*-Value	Cohen’s d
Perceived Stress Scale	0.581 (0.537, 0.625)	0.022	26.045	<0.001 ***	2.04
General Psychological Health	0.224 (0.194, 0.254)	0.015	14.667	<0.001 ***	1.21

Note: Separate linear regression analyses were conducted for perceived stress and general psychological health, with cyberbullying victimization as the predictor variable in each model. B = unstandardized regression coefficient; SE = standard error; CI = confidence interval. *** *p* < 0.001. Cohen’s d represents the effect size, with 0.2, 0.5, and 0.8 indicating small, medium, and large effects, respectively.

**Table 6:** Mediation analysis of perceived stress on relationship between cyberbullying victimization and general psychological health (*n* = 562).

Path	B (95% CI)	SE	t	*p*-Value
a (Cyberbullying Victimization → Perceived Stress)	1.876 (1.516, 2.236)	0.183	10.241	<0.001 ***
b (Perceived Stress → General Psychological Health)	0.581 (0.537, 0.625)	0.022	26.045	<0.001 ***
c (Cyberbullying Victimization → General Psychological Health)	1.451 (0.958, 1.944)	0.251	5.783	<0.001 ***
c’ (Cyberbullying Victimization → General Psychological Health, direct)	0.362 (−0.088, 0.812)	0.229	1.580	0.115
Indirect effect (a*b)	1.089 (0.874, 1.304)	0.110	-	<0.001 *

Note: * *p* < 0.05, *** *p* < 0.001; Path a represents the effect of cyberbullying victimization on perceived stress. Path b represents the effect of perceived stress on general psychological health. Path c represents the total effect of cyberbullying victimization on general psychological health, without controlling for the mediator (perceived stress). Path c’ represents the direct effect of cyberbullying victimization on general psychological health, after controlling for the mediator. The indirect effect (a*b) represents the mediated effect of cyberbullying victimization on general psychological health through perceived stress. B = unstandardized regression coefficient; SE = standard error; CI = confidence interval. A mediation analysis was conducted using the PROCESS macro for SPSS (Model 4, with 5000 bootstrap samples).

## Data Availability

The data presented in this study are available on request from the corresponding author. They are not publicly available due to the sensitive nature of the information collected from minors and the need to protect their privacy and confidentiality.
